# Winter Shoes

**Published:** 1871-02

**Authors:** 


### WINTER SHOES.

In the sloshy weather of winter, when the roads and streets are covered with mud or half-melted snow, India rubber shoes are a perfect protection; but to wear them longer than an hour or two, especially if the person is not in continuous exercise, has the certain effect to make the feet cold and clammy, thus preparing the system for colds, croups, and inflammation of the lungs.

Workmen and business men must be on their feet more or less all day; and to have the feet dry and warm is essential to health and comfort, and even to life itself.

The soles of shoes can be made impervious to dampness if they alone are soaked for twenty-four hours in kerosene oil, and are then allowed to dry thoroughly. Let the sole be deep enough in the oil to cover the top of it, so that the oil may sink in among the stiches, and fill in the seams of the sole and upper leather; then the upper leather may be polished with blacking, but it could not be made to shine as well if soaked in oil, nor is it desirable to have the upper leather impervious, for then it would be no better than India rubber.

All persons should have cork soles in their shoes, the cork being covered on the side next the foot with Canton flannel; but it would be well to take them out every night, and place them where they will be well aired and dried. Another benefit of cork soles is, they save about ninety per cent. of darning. Nine shoemakers out of ten fail to rasp off the pegs and nails on the inside of the sole, the result being in many cases that holes are worn in the stockings in twenty-four hours after their first wearing. But even if the wooden pegs are ever so well rasped, or the iron pegs clinched, still wood and iron are harder than leather, have no yield, and wear holes in the best stockings in a short time; and as a large portion of the time of our industrious wives at the close of the week, and especially on Saturday nights, is spent in darning stockings, any suggestion that saves them work is worth consideration.

